# Distribution of Microbial Arsenic Reduction, Oxidation and Extrusion Genes along a Wide Range of Environmental Arsenic Concentrations

**DOI:** 10.1371/journal.pone.0078890

**Published:** 2013-10-31

**Authors:** Lorena V. Escudero, Emilio O. Casamayor, Guillermo Chong, Carles Pedrós-Alió, Cecilia Demergasso

**Affiliations:** 1 Centro de Investigación Científico Tecnológico para la Minería-CICITEM, Universidad Católica del Norte, Antofagasta, Chile; 2 Centro de Biotecnología, Universidad Católica del Norte, Antofagasta, Chile; 3 Departamento de Ciencias Geológicas, Universidad Católica del Norte, Antofagasta, Chile; 4 Biogeodynamics & Biodiversity Group, Centre d’Estudis Avançats de Blanes, CEAB-CSIC, Blanes, Spain; 5 Institut de Ciències del Mar, CSIC, Barcelona, Spain; University of Exeter Medical School, United Kingdom

## Abstract

The presence of the arsenic oxidation, reduction, and extrusion genes *arsC*, *arrA*, *aioA*, and *acr3* was explored in a range of natural environments in northern Chile, with arsenic concentrations spanning six orders of magnitude. A combination of primers from the literature and newly designed primers were used to explore the presence of the *arsC* gene, coding for the reduction of As (V) to As (III) in one of the most common detoxification mechanisms. Enterobacterial related *arsC* genes appeared only in the environments with the lowest As concentration, while Firmicutes-like genes were present throughout the range of As concentrations. The *arrA* gene, involved in anaerobic respiration using As (V) as electron acceptor, was found in all the systems studied. The As (III) oxidation gene *aioA* and the As (III) transport gene *acr3* were tracked with two primer sets each and they were also found to be spread through the As concentration gradient. Sediment samples had a higher number of arsenic related genes than water samples. Considering the results of the bacterial community composition available for these samples, the higher microbial phylogenetic diversity of microbes inhabiting the sediments may explain the increased number of genetic resources found to cope with arsenic. Overall, the environmental distribution of arsenic related genes suggests that the occurrence of different ArsC families provides different degrees of protection against arsenic as previously described in laboratory strains, and that the glutaredoxin (Grx)-linked arsenate reductases related to Enterobacteria do not confer enough arsenic resistance to live above certain levels of As concentrations.

## Introduction

Arsenic toxicity is a major problem in many parts of the world where drinking water supplies are heavily contaminated [1], [2]. This has prompted considerable research on biogeochemical and microbiological processes that control the distribution and mobilization of As in aquatic environments [3], [4]. Despite the fact that arsenic transformations are carried out by a large variety of microorganisms, the number of different genes involved seems to be limited to a few types that are widely distributed across phylogenetic lineages. Therefore, there is a possibility of detecting the presence of such genes in the environment with a limited number of primers [5], [6]. This possibility is attractive because the monitoring of the presence of As-related genes may help in detecting and managing As contamination events.

The genes used by prokaryotes to cope with As fit into five groups: (i) As(V) detoxifying reduction *ars* operon, (ii) As(V) *arr* respiratory reduction operon, (iii) As(III) oxidation *aio* operon, (iv) As(III) extrusion genes *acr3*, and (v) methylation genes not specific for arsenic [7], [8]. Many bacteria can detoxify As by the Ars system —plasmid or chromosome encoded—, which is widespread in nature and has been extensively studied [9], [10]. The key enzyme is the *arsC* product that reduces As(V) to As(III). The latter can then be extruded out of the cell by the pump coded by *arsB*. The operon includes up to five genes, some of them are regulators and the *arsC* gene is almost always present [11]. Three different ArsC prokaryotic families have been defined based on the protein structures, the reduction mechanisms, and the location of the catalytic cysteine residues: i) the glutathione (GSH)/glutaredoxin (Grx)-coupled class) (plasmid R773 of *Escherichia coli*) [12], ii) the thioredoxin (Trx)/thioredoxin reductase (TrxR)-dependent class (plasmid pl258 of *Staphylococcus aureus*) [13] and iii) the mycothiol (MSH)/mycoredoxin (Mrx)-dependent class found in Actinobacteria [14]–[16]. Kinetics data of arsenate reduction have shown a higher catalytic efficiency in thyoredoxin (Trx)- than in glutaredoxin (Grx)-linked arsenate reductases [16]. Some authors have suggested that these families arose independently [7], [12]. Conversely, a single early origin of the *arsC* gene and subsequent sequence divergence has also been proposed [17]. In any case, the families are sufficiently different that they require different primer sets to be fully targeted. Thus, besides the three sets of primers described in the literature [18]–[20], here we developed three additional sets to cover the full genetic diversity of the *arsC* gene.

Another widespread set of genes involved in As reduction is the *arr* operon (As respiration). The *arr* and *ars* operons are not mutually exclusive and the presence of both has been shown in *Shewanella* sp. [18]. Bacteria with the *arr* operon use As(V) as electron acceptor and reduce it to As(III), a more toxic and more mobile form [18]. Some bacteria, however, respire both As(V) and sulfate, precipitating As sulfides such as realgar and orpiment, thus providing a potential bioremediation mechanism [21]. Arsenic sulfide-precipitating microorganisms have been found in several contaminated environments in northern Chile by means of most probable number estimations and isolations in pure culture [22]. The functional gene for arsenate respiration, *arrA*, is highly conserved [7]. Three primer sets have been designed in the literature [6], [23], [24] and they were all tested in the present work.

The microbial oxidation of arsenite is also a widespread mechanism in soil and water systems containing As. Many bacteria oxidize arsenite as a step in detoxification, since As(V) is less toxic than As(III). But some bacteria are able to use As(III) as a source of electrons for respiration. Recently, a common nomenclature has been proposed for homologous arsenite oxidizing genes [25]. Thus, for example, *aioA* (arsenite oxidation) stands for the formerly named *aoxB*, *asoA* and *aroA*. The genes for aerobic arsenite oxidation are widely distributed in the bacterial domain and two different primer sets have been designed in the literature, which were used here to detect these genes [5]. In addition, a new gene family, arxA, has been identified through mutagenesis and physiological experiments that is required for chemoautotrophic growth with arsenite coupled to nitrate respiration [26].

The *acr3* gene product is a pump that extrudes As(III) out of the cells [27], [28]. The function of this protein is analogous to that of *arsB*, a component of the more common detoxifying operon. Two families have been identified and the corresponding primers have been published [29] and were used here to detect their presence.

Finally, methylation has been proposed as an additional detoxification strategy [30]. An arsenite S-adenosylmethionine methyltransferase (*arsM*) has been characterized in *Rhodopseudomonas palustris* and belongs to the UbiE/Coq5 C-methyltransferase family [31]. The *arsM* gene has been detected in more than 120 Bacteria and in about 15 Archaea [32]. However, the enzyme is not specific for the As(V) to As(III) reduction step [7] and, therefore, we have excluded this gene from the present study.

Most of the primers mentioned above have not been tested in natural environments, or have been tested in one or a few natural samples only [5], [19], [24], [33], covering a very limited range of As concentrations. In addition, some of the primers do not target the whole diversity of known genes [19], [20], [34]. In the Andean altiplano (including areas of Chile, Argentina, Bolivia and Peru), As contamination is a natural process that has occurred for long periods of time. Many people drink water with toxic As levels, even up to 0.6 mg L^−1^ [35]. Some environments in this region show the largest As concentrations recorded in natural systems [22]. We, therefore, tested 11 [5], [6], [18]–[20], [23], [24], [29] of the 19 available primers [26], [36]–[40] in a series of environments including thermal springs, stream waters, lagoons, mats lining hot springs and several types of sediments that, besides being conveniently close in space, offered a range of As concentrations spanning six orders of magnitude.

## Materials and Methods

### Site description

**Figure 1:**
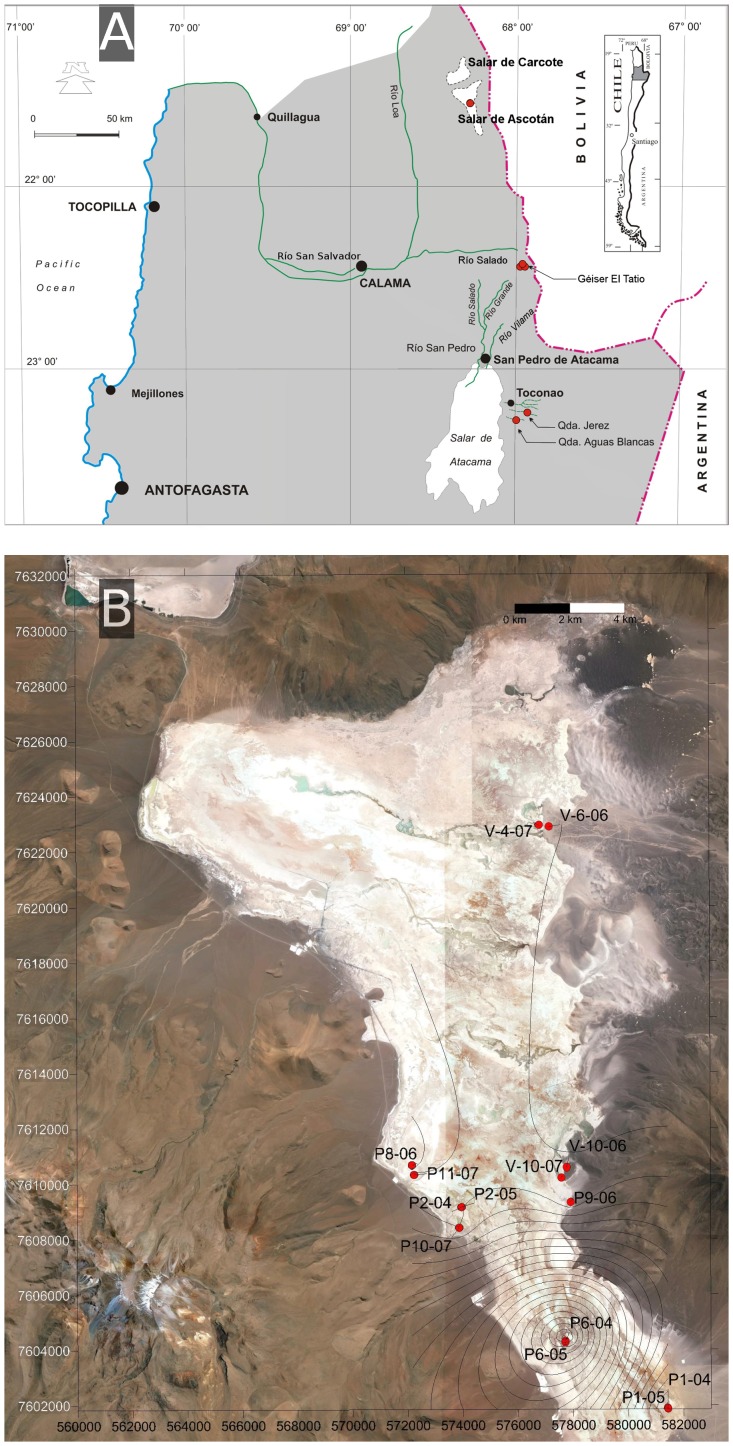
Maps of the sampling location. (A) Map of northern Chile showing the areas where samples were taken: 24 in Ascotán, three in El Tatio, and 2 in Atacama. (B) Modified satellite image of Salar de Ascotán showing the location of sampling spots. The contours of As level in water was constructed using the Surfer software program (v.7.0, Golden Software, USA). Point P6 had the highest concentration in the water. Concentrations in sediments were higher in all cases.

Table 1 shows a summary of the geographical location, altitude and other environmental parameters for the systems studied. We chose different As-rich environments located between UTM 19 K 572171 and 603405 South and between 7427145 and 7622927 West (Fig. 1 A and B). Most sites were above 3700 m of altitude. These included several samples from the main Andean salts deposits (Salar de Ascotán), from a geothermal geyser field (El Tatio) and from incoming rivers (i.e. “quebradas”) to Salar de Atacama (Quebrada de Jere and Quebrada Aguas Blancas). No specific permissions were required for sampling in Salar de Ascotán, Quebrada de Jere, Quebrada Blanca and El Tatio. For sampling in El Tatio we informed the Comunidad Indígena de Toconce and Socaire. All these systems shared the presence of As in large or small concentrations (see Table 1). Salar de Ascotán was studied in more detail because the As concentrations were the highest found in the area. Salar de Ascotán is an athalassohaline environment located at the bottom of a tectonic basin surrounded by volcanic systems in east-west direction, including some active volcanoes over 5000 m high, with the highest altitudes close to 6000 m. Climate is characterized by large daily thermal oscillations. High solar irradiance, and strong and variable winds cause intense evaporation (about 4.5 mm day^−1^) while precipitation is about 120 mm year^−1^ [41]. Water inputs include surface drainage from the snow fields on volcanoes, ground waters with a strong geothermal component and spring waters commonly reaching 23°C to 25°C. The saline crusts are mainly composed of chlorides (halite) and sulfates (gypsum) with important borate ore deposits composed mostly of ulexite with significant amounts of As sulfide minerals. This system exhibits high spatial and temporal variability with water salinities ranging from freshwater to salt-saturated brines [42].

**Table 1:** Systems sampled, date, coordinates, altitude and some physicochemical parameters.

Sample code	Location	Sample type	Year	UTM coordinates Grid 19K		Altitude (m)	Total As (mg L^−1^) (mg Kg^−1^)	Temperature °C	pH	Salinity (g L^−1^)	Conductivity (mS cm^−1^)	TDS (mg L^−1^)	Dissolved Oxigen (mg L^−1^)	DAPI (cell mL^−1^) (cell g^−1^)
Jere*	Atacama	Stream	2007	7435185	603405	2485	0.04	17	7.2	0.1	0.3	145	7,8	2.7E+06
V-4*	Ascotán	Spring	2007	7622927	576823	3748	0.7	20.4	8.6	2.1	4.1	2120	7,8	4.9E+06
V-6	Ascotán	Spring	2006	7622870	577180	3748	0.9	16	nd	0.5	1.0	493	10,8	1.2E+06
V-10*	Ascotán	Spring	2007	7610212	577586	3748	1.0	24.5	8.1	3	5.6	2950	8	9.3E+05
V-10	Ascotán	Spring	2006	7610573	577782	3748	1.6	16	8.0	1.5	2.9	1390	8,8	1.5E+06
P9*	Ascotán	Brine	2006	7609328	577921	3748	3.4	0	8.3	1.8	3.9	1750	8,9	3.9E+06
P1-04*	Ascotán	Brine	2004	7601844	581436	3748	3.5	15.5	6.9	0.1	0,3	143	n.d	7.7E+05
P11*	Ascotán	Brine	2007	7610325	572247	3748	4.0	17.4	8.2	3.5	6.6	3410	6,4	2.6E+06
Aguas Blancas*	Atacama	Stream	2007	7427145	603446	2485	4.0	16	8.14	1	1.9	963	4,9	5.0E+05
P1-05	Ascotán	Brine	2005	7601876	581420	3748	4.4	10	7.8	33	41.4	31500	n.d	6.7E+05
Tatio-SL-2	Geiser del Tatio	Geiser	2007	7530555	602449	4280	6.0	56	2.5	6.9	12.6	6720	2,3	9.0E+05
P2-05	Ascotán	Brine	2005	7609154	573967	3748	6.5	14	8	76	79.1	92300	n.d	5.0E+05
P10-07*	Ascotán	Brine	2007	7608410	573881	3748	10.0	4.9	8.4	6.2	11.5	6003	7,1	8.1E+06
P2-04*	Ascotán	Brine	2004	7609154	573971	3748	10.6	16	8.5	0.7	1.4	659	n.d	7.0E+05
Tatio-SL-1*	Geiser del Tatio	Geiser	2007	7530388	602108	4278	14.0	52	3.3	5.1	9.1	4830	2,8	4.2E+06
P8	Ascotán	Brine	2006	7610653	572171	3748	28.0	3	8.3	10.5	18.5	10200	7,5	4.3E+07
Tatio-SL-3*	Geiser del Tatio	Geiser	2007	7530214	601730	4284	36.0	78.3	6.7	13.2	22.4	11800	1,5	1.8E+06
P6-05*	Ascotán	Brine	2005	7604253	577706	3748	183	17	7.2	309	193.4	628000	n.d	1.6E+05
P6-04*	Ascotán	Brine	2004	7604308	577712	3748	212	17.5	7.4	7.1	12.4	6940	n.d	2.6E+05
V-6	Ascotán	Spring sediment	2006	7622870	577180	3748	370							5.5E+07
P9	Ascotán	Brine sediment	2006	7609328	577921	3748	610							6.0E+07
P3-05*	Ascotán	Brine sediment	2005	7609154	574012	3748	781							2.3E+06
P8	Ascotán	Brine sediment	2006	7610653	572171	3748	800							5.1E+07
V-10	Ascotán	Spring sediment	2006	7610573	577782	3748	950							1.9E+08
P4-05*	Ascotán	Brine sediment	2005	7609035	573669	3748	1210							3.0E+06
P4-04	Ascotán	Brine sediment	2004	7609029	573679	3748	1280							1.8E+06
P3-04	Ascotán	Brine sediment	2004	7609154	573971	3748	1690							1.3E+06
P7-05	Ascotán	Brine sediment	2005	7609035	577770	3748	6504							6.8E+05
P7-04*	Ascotán	Brine sediment	2004	7604308	577712	3748	9440							5.8E+05

* Samples analyzes by DGGE.

### Sample collection and processing

Four sampling expeditions to Salar de Ascotán were conducted in November 2004, August 2005, June 2006 and April 2007 (Table 1). Eighteen sampling sites were selected within the basin (P1 to P11) and three thermal springs (“vertientes” V4, V6 and V10) for sampling both water and sediments at different times (Table 1 and Fig. 1 A and B). Overall, 29 samples were analyzed. Temperature and pH were measured with a pH meter Orion model 290. A conductivity meter Orion model 115 was used for measuring salinity, conductivity and total dissolved solids. Oxygen was measured with a Thermo Orion model 9708. Water samples were kept in polyethylene 2-L bottles in an icebox until further processing during the next 24 hr. Samples for total cell counts were fixed *in situ* with 4% formaldehyde (vol/vol, final concentration) overnight at 4°C. Counts were done by epifluorescence microscopy staining with a DNA-specific dye, 4′, 6-diamidino-2-phenylindole (DAPI) with a Leica DMLS epifluorescence microscope. Arsenic concentrations were measured using Hydride generation atomic absorption spectroscopy (HG-AAS), directly on the brine samples and after acid digestion of the sediment samples [22].

### Total DNA extraction

DNA collection and extraction were done as described before [22]. Between 800 and 1000 mL of water was filtered through 0.2 µm polycarbonate membranes (Nuclepore). The filters were stored at −20°C in 1 mL lysis buffer (50 mM Tris–HCl pH = 8.3, 40 mM EDTA and 0.75 M sucrose) [43]. For sediment samples, nucleic acids were extracted from 25 to 50 g sediment (wet weight), and were actively vortexed in a salt solution (PBS buffer 1x, Tween 20 at 10% v/v) at 120 rpm. The supernatant was filtered and processed as previously done with water samples. The filters were incubated with lysozyme and proteinase K [43], and genomic DNA was extracted with a High Pure Template Preparation Kit (Qiagen).

### Detoxifying arsenate reductase *arsC* genes

**Figure 2:**
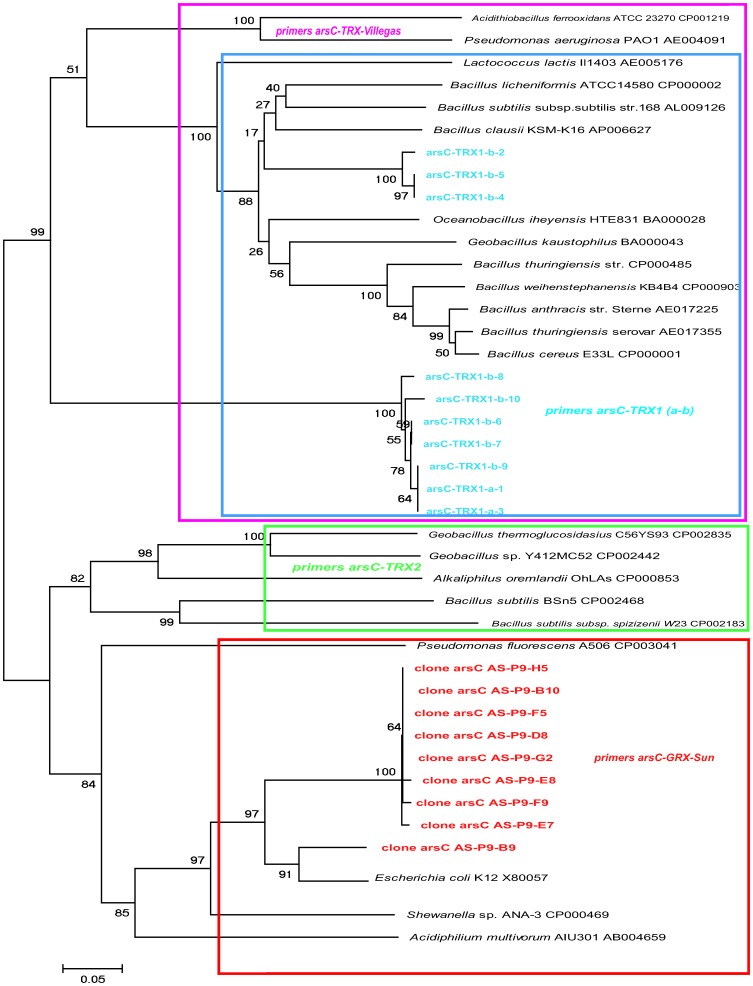
Tree of the *arsC* genes used to select the sequences for primer design. The sequences targeted by each reported and designed primer set are enclosed in colored rectangles. See Table 2 for primer sequences. The tree was constructed by Neighbor-joining. Bootstrap values for 500 replicates are indicated at the nodes.

The *arsC* gene was targeted using six primer sets (Table 2). The sequences targeted by each primer set are shown in Figure 2. The first primer set (arsC*-*Grx-Sun) contained a mixture of primers amlt-42-f/amlt-376-r and smrc-42-f/smrc-376-r [19]. The PCR amplification conditions were as follows: 95°C for 3 min, 40 cycles of 95°C for 15 s denaturation, 60°C for 15 s annealing, and 72°C for 15 s elongation, in a primer mixture of 0.25 µM each. These primers amplify a fragment of 353 bp of the *arsC* gene. The second set (arsC-Grx-Saltikov) consisted of primers Q-arsC-f1 and Q-arsC-r1 [18]. The PCR amplification conditions were as follows: 95°C for 10 min, 40 cycles of 95°C for 30 s (denaturation), 60°C for 1 min (annealing), and 72°C for 30 s (elongation), at 0.5 µM each primer. The third set (arsC-Trx-Villegas) consisted of primers arsCGP-Fw and arsCGP-Rv [20] for the amplification of the ArsC thioredoxin dependant mechanism (arsC-Trx). The PCR program for these primers was 5 min denaturation at 95°C followed by 35 cycles of 95°C for 90 s, 48°C for 90 s, and 72°C for 2 min, and finally 5 min extension at 72°C. The concentration of primers was 0.3 µM [20].

**Table 2:** Primer sets used in this study for PCR amplification of several genes involved in the arsenic cycle.

Targeted gene	Primer Set^1^	Primer name^2^	Primer sequence (5′ – 3′)	Amplicon lenght (bp)	Reference (system studied)
Arsenate respiratory reductase	arrA 1	arrAf	AAG GTG TAT GGA ATA AAG CGT TTG TBG GHG AYT T	160–200	[6]
		arrAr	CCT GTG ATT TCA GGT GCC CAY TY V GGN GT		(Haiwee Reservoir and *Shewanella* sp. ANA-3)
	arrA 2	AS1f	CGA AGT TCG TCC CGA THA CNT GG	625	[24]
		AS1r	GGG GTG CGG TCY TTN ARY TC		(Cambodian Sediments)
		AS2f (nested)	GTC CCN ATB ASN TGG GAN RAR GCN MT		
	arrA 3	HAArrA-D1f	CCG CTA CTA CAC CGA GGG CWW YTG GGR NTA	500	[23]
		HAArrA-G2r	CGT GCG GTC CTT GAG CTC NWD RTT CCA CC		(Mono Lake and Searles Lake, California)
Arsenate reductase	arsC-Grx-Sun mix	amlt-42-f	TCG CGT AAT ACG CTG GAG AT	334	[19]
		amlt-376-r	ACT TTC TCG CCG TCT TCC TT		(Bacterial strain and plasmid)
		smrc-42-f	TCA CGC AAT ACC CTT GAA ATG ATC		
		smrc-376-r	ACC TTT TCA CCG TCC TCT TTC GT		
	arsC-Grx-Saltikov	Q-arsC-f1	GAT TTA CCA TAA TCC GGC CTG T	200–300	[18]
		Q-arsC-r1	GGC GTC TCA AGG TAG AGG ATA A		(*Shewanella* sp. strain ANA-3)
	arsC-Trx-Villegas	arsCGP-Fw	TGC TG ATTT AGT TGT TAC GC	353	[20](As-resistant bacteria isolated from contaminated soil)
		arsCGP-Rv	TTC CTT CAA CCT ATT CCC TA		
	arsC-Trx1a	arsC 4f	TCH TGY CGH AGY CAA ATG GCH GAA G	300–400	This study
		arsC 4r	GCN GGA TCV TCR AAW CCC CAR TG		
	arsC-Trx1b	arsC 5f	GGH AAY TCH TGY CGN AGY CAA ATG GC	300–400	This study
		arsC 5r	GCN GGA TCV TCR AAW CCC CAR NWC		
	arsC-Trx2	arsC 6f	CAC VTG CMG RAA DGC RAR RVV DTG GCTCG	300–400	This study
		arsC 6r1	YKY CRY CBR YVA DRA TCG G		
		arsC 6r2	TTR WAS CCN ACG WTA ACA KKH YYK YC		
		arsC 6r3	YYV HWY TSK TST TCR YKR AAS CC		
Arsenite transporter	acr3 1	dacr1-F	GCC ATC GGC CTG ATC GTN ATG ATG TAY CC	750	[29]
		dacr1-R	CGG CGA TGG CCA GCT CYA AYT TY TT		
	acr3 2	dacr5-F	TGA TCT GGG TCA TGA TCT TCC CVA TGM TGV T	750	
		dacr4-R	CGG CCA CGG CCA GYT CRA ARA ART T		
Arsenite oxidase	aioA 1	aroA #1F	GTS GGB TGY GGM TAY CAB GYC TA	500	[5](Arsenic-impacted soils,
		aroA #1R	TTG TAS GCB GGN CGR TTR TGR AT		
	aioA 2	aroA #2F	GTC GGY YGY GGM TAY CAY GYY TA	500	sediments and
		aroA #2R	YTC DGA RTT GTA GGC YGG BCG		geothermal mats, USA)

1 Code used in the present paper.

2 Name used in the original publication.

The next three sets of primers (arsC-Trx1a, arsC-Trx1b, arsC-Trx2) were designed in the present study. We collected the known *arsC* sequences from the literature [12] and from GenBank (retrieved from NCBI) and we also searched the available genomes of the predominant phylotypes in the studied environment. It appeared that Proteobacterial sequences were well covered by the *arsC-*Grx-Sun [19]. Some Firmicutes sequences, on the other hand, were not covered by any primer set. Sequences were aligned using Clustal X [44] and manually corrected. The aligned *arsC* sequences were used to construct a baseline phylogenetic tree using neighbor-joining (NJ), maximum parsimony (MP) and maximum likelihood (ML) methods (Figure 2). The tree was constructed using the Molecular Evolutionary Genetic Analysis (MEGA) 4 (http://www.megasoftware.net) software with 500 bootstrap replicates. To design the arsC-Trx1a and arsC-Trx1b sets of primers we used *arsC* sequences from the following bacteria: *Bacillus thuringiensis, Bacillus cereus, Geobacillus kaustophilus, Bacillus clausii, Oceanobacillus iheyensis* and *Bacillus halodurans* (Fig. 2 and Fig. S1 A). The sequences used for designing primer set arsC-Trx2 were *Geobacillus thermoglucosidasius, Geobacillus* sp., *Alkaliphilus oremlandii* OhILAs and *Bacillus subtilis* (Fig. 2 and Fig. S1 B). The Sequence Manipulation Suite (SMS) (http://bioinformatics.org/sms2/index.html) was used to verify the properties of each designed primer.

Primers arsC-Trx1a and arsC-Trx1b amplify a 300–400 bp fragment of the arsenate reductase gene (Table 2). The PCR conditions with the arsC-Trx1a set were 5 min denaturation at 95°C, followed by 35 cycles of 95°C for 90 s, 46.7°C for 90 s, and 72°C for 3 min, and finally 5 min extension at 72°C. The PCR conditions with the arsC-Trx1b set were the same as for arsC-Trx1a set but using a different annealing temperature (60°C). For the primer set arsC-Trx2 we tried one forward primer and three reverse primers. The reverse primer arsC6r2 resulted in the best outcome. The PCR conditions were 5 min denaturation at 95°C, followed by 35 cycles of 95°C for 90 s, 54.5°C for 90 s, and 72°C for 3 min, and finally 5 min extension at 72°C. The concentration of primer was 0.3 µM. The PCR representative products (14) were purified using a QIAGEN PCR cleanup kit according to manufacturer instructions and sent for sequencing to Macrogen (Macrogen Inc., Korea, www.macrogen.com) to confirm that the amplicon obtained with the newly designed primers were bona fide *arsC* gene fragments. Sequences for the *arsC* genes were sent to BLAST (Basic Local Alignment Search Tool) algorithm [45] at the National Center for Biotechnology Information (NCBI, http://www.ncbi.nlm.nih.gov/BLAST/) for BLASTX and BLASTN searches to determine the closest relative in the database. The retrieved sequences ranged from 86% to 96% identity to *arsC* sequences available in GeneBank.

### Dissimilatory arsenate reductase *arrA* genes

Three primer sets were used for the amplification of the As respiratory gene *arrA*. The first primer set arrA1 included the arrAf and arrAr primers [6] that amplify a ∼160–200 bp fragment of the gene. The optimized PCR conditions included incubation at 95°C for 10 min, followed by 40 cycles of 95°C for 15 s, 50°C for 40 s, and 72°C for 1 min [6]. The concentration of each primer in a single reaction was 0.3 µM.

The second primer set was arrA2, used in a nested PCR approach with primers AS1f, AS1r and AS2f [24]. This primer set was designed after comparison of conserved regions in the *arrA* genes from *Bacillus selenitireducens, Chrysiogenes arsenatis, Shewanella* sp. strain ANA-3, *Desulfitobacterium hafniense* DCB2, and *Wolinella succinogenes.* The nested-PCR with the primer combination AS2f and AS1r yielded a 625 bp product. The first PCR step was carried out by using a 5 min denaturation step at 94°C, followed by 35 cycles of a 30 s denaturation at 94°C, primer annealing of 30 s at 50°C, and a 1min extension at 72°C. The second PCR amplification (nested) was done with primers AS2F and AS1R using the first PCR product as template. Nested PCR began with a 2 min denaturation step at 94°C, followed by 30 cycles of a 30 s denaturation at 94°C, primer annealing of 30 s at 55°C, and a 1min extension at 72°C. The concentration of primers in each PCR was 0.3 µM.

Finally, the third primer set for gene *arrA* was arrA3 (HAArrA-D1f and HAArraA-G2r) that generated a 500 bp PCR product [23]. The PCR conditions were initial denaturation at 95°C for 5 min, followed by 40 cycles of denaturation at 94°C for 30 s, primer annealing at 53.5 °C for 30 s, and extension at 72°C for 30 s, with an additional step at 85° C for 10 s, each primer at the concentration of 0.3 µM.

### Arsenite oxidation *aioA* gene

The PCR amplification of *aioA* was carried out using two sets of degenerate primers (aioA1 and aioA2) (Table 2) to amplify approximately 500 bp and bind at nucleotide positions 85–107 and the reverse primers bind at nucleotide positions 592–614 and 601–621, respectively, in the *aioA* sequence of *Rhizobium* sp. str. NT26 [5]. The first set aioA1 consisted in aioA-1F and aioA-1R and the PCR conditions were 95°C for 4 min followed by nine cycles of 95°C for 45 s, 50°C (decreased by 0.5°C after each cycle) for 45 s, 72°C for 50 s, followed by 25 cycles of 95°C for 45 s, 46°C for 45 s, and 72°C for 50 s, and a final extension of 72°C for 5 min. The second set of primers aioA 2 were aioA-2F and aioA-2R, and the PCR conditions for primer aioA2 were 90°C for 3 min, followed by 40 cycles of 92°C for 1 min, 50°C for 1.5 min, 72°C for 1 min and final extension of 72°C for 5 min. The concentration of each primer was 0.3 µM.

### Arsenite transport genes acr3

The amplification of *acr3-1* and *acr3-2* genes were done with two pairs of degenerate primers (0.3 µM), dacr1F and dacr1R for *acr3-*1 and dacr5F and dacr4R for *acr3-*2 as described by Achour et al. (2007) [29]. The cycling conditions for all PCRs consisted of 5 min of denaturation at 94°C followed by 35 cycles of 45 s of denaturation at 94°C, 45 s of annealing at 57°–52°C with a 0.5°C decrement per cycle during the first 10 cycles, and 30 s of primer extension at 72°C. This was followed by an extension reaction at 72°C for 7 min.

The PCR ingredients for all primer sets included 10% (v/v) DMSO and 0.5U of Taq DNA Polymerase (Invitrogen, Tech-Line). The presence of PCR amplification products was verified by electrophoresis in 1% agarose gels stained with ethidium bromide. The DNA sample reference 595 obtained from a coastal marine environment in Blanes Bay Microbial Observatory was used as a negative control. *Fusibacter* sp. 3D3RP8 (FR873490) obtained in those studies was used as positive control for reduction and negative control for oxidation, and *Herminimonas arsenicoxidans* was used as negative control for reduction and positive control for oxidation.

### Denaturing gradient gel electrophoresis (DGGE)

The extracted genomic DNA of 16 of the samples was used as target in the PCR to amplify 16S rRNA genes. Bacterial fragments were amplified with the primer set 358FGC-907R [43] and DGGE was performed as described previously [43]. The bands were carefully excised from the DGGE gel with a razor blade under UV radiation for further identification as has been reported before [46]. Sequencing was carried out directly on PCR products with the 341F primer in Macrogen. Sequences were deposited in GenBank (accession numbers AB844273-AB844320).

The identity and similarity to the nearest neighbor of DGGE band sequences were obtained using the BLAST algorithm [45]. The relative abundance of the major groups was calculated adding the number of the DGGE bands with sequences affiliated to those groups after the phylogenetic analysis.

### Construction of 16S clone libraries

Samples P9 (water) and P4 (sediment) from Salar de Ascotán were selected for clone libraries because they had some the lowest (3.4 mg L^−1^) and the highest (1,210 mg Kg^−1^) total arsenic concentrations, respectively. 16S rRNA genes were amplified by PCR with the universal primers 27F Mod (5′-AGR(AG) GTT TGA TCM(AC) TGG CTC AG-3′) and 1492R Mod (5′-GGY(CT) TAC CTT GTT AY G ACT T-3′) and cloned into pCR2.1 vector with the TOPO TA cloning kit Catalog #4500-01 (Invitrogen Carlsbad, California) and transformed into TOP10 chemically competent cells. The transformed cells were grown on Luria-Bertani plates containing 50 mg of Kanamycin mL^−1^, 20 mg of 5-bromo-4-chloro-3-indolyl-β-D-galactopyranoside (XGal) m L^−1^, as recommended by the manufacturer and incubated overnight at 37°C. Cloning and sequencing were carried out as previously described [43]. Clones were analyzed with database nucleotide BLAST against Nucleotide collection (nr/nt). Operational Taxonomic Units (OTUs) were determined by Restriction Fragments Length Polimorphism (RFLP) with enzyme HAE III. All sequences belonging to the same OTU were found to be >97% identical. Sequences were deposited in GenBank under accession numbers FM879025 to FM879135.

## Results

Geographical location, physicochemical data and biological parameters for all sites sampled are shown in Table 1. The sampling spots from Salar de Ascotán are shown in Figure 1 B. The samples were obtained at different years and seasons and the temperatures ranged between 0°C and 25°C, except for samples from El Tatio geyser field, with temperatures ranging between 52°C and 78°C. Salinity values were between 0.1 and 309 g L^−1^, conductivity ranged between 0.3 and 193.4 mS cm^−1^ and total dissolved solids (TDS) between 143 and 628000 mg L^−1^. Sample P6, collected on August 9, 2005, consisted of brine with the highest values of salinity, conductivity, and TDS. Dissolved oxygen ranged between 5.0 and 10.8, except for samples from El Tatio geyser field SL1 and SL2 (DO 2.8 – 1.5, respectively). We found total arsenic concentration values between 0.04 and 212 mg L^−1^ in water samples, and between 370 and 9440 mg Kg^−1^ in the sediments. These are some of the highest values of total arsenic reported so far [1]. The concentrations of DAPI-stained cells ranged between 1.6×10^5^ and 4.3×10^7^ cells mL^−1^ in the water samples and between 5.8×10^5^ and 1.9×10^8^ cells g^−1^ in the sediments (Table 1).

**Figure 3:**
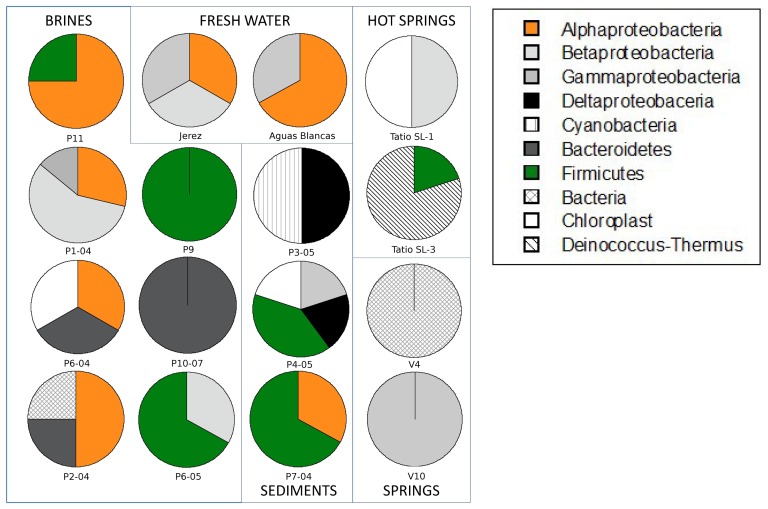
Relative abundance of different phylogenetic groups of bacteria in 16 (indicated by an asterisk in Table 1) of the samples analyzed by DGGE.

**Figure 4:**
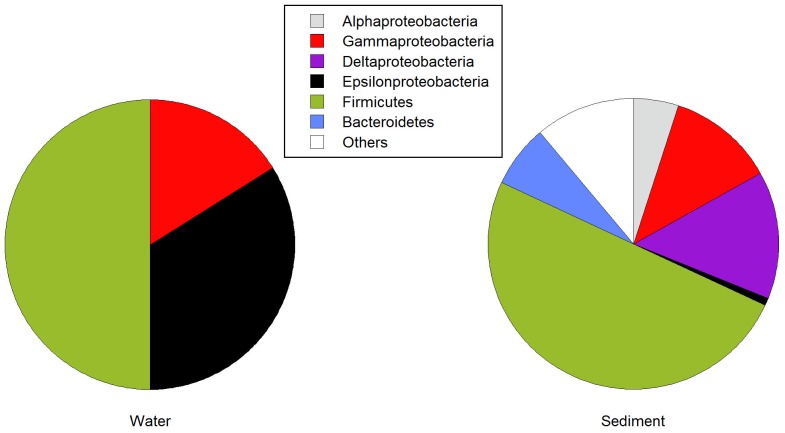
Relative abundance of major taxonomic groups in clone libraries from two selected samples (P9 water and P4 sediment) with different total arsenic concentrations from Salar de Ascotán.

The relative abundance of the major bacterial groups was analyzed by DGGE (Fig.3, Table S1). There was considerable variation among the 16 samples (indicated by asterisks in Table 1). For example, one sample was dominated by Bacteroidetes and another by Deinococcus-Thermus, even though neither one of this groups was abundant in general. Overall, however, Proteobacteria and Firmicutes were the dominant groups (Fig. 3, Table S1). Two samples, one water with low As and one sediment with high As, were selected to build clone libraries as examples (Fig. 4). Firmicutes were clearly dominant in both, followed by Proteobacteria. The particular Proteobacterial classes in each one, however, were different: gamma and epsilon in the water sample and alpha, gamma and delta in the sediment.

### Distribution of genes involved in As redox transformations and transport

We used a total of 11 primer combinations to explore the presence of As related genes in 19 water and 10 sediment samples from Salar de Ascotán, El Tatio geyser field, and two desert streams in Atacama (Fig. 1, Table 1).

**Figure 5:**
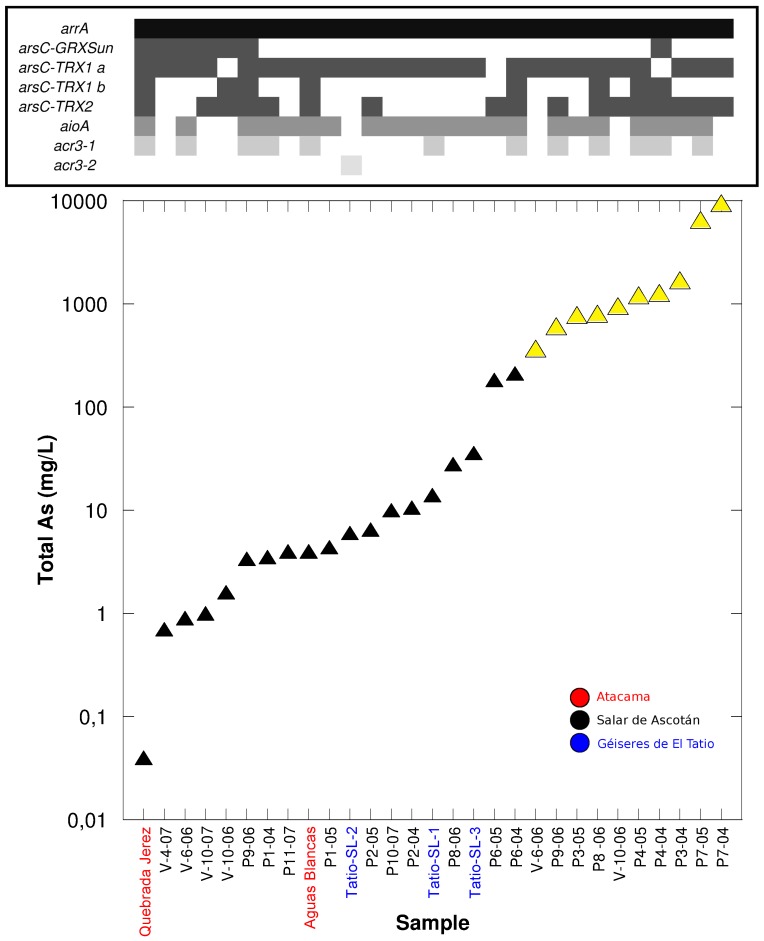
Total [As] in the natural environments analyzed (black triangles for water samples [mg/L] and yellow triangles for sediment samples [mg/Kg]), and range of concentrations where the arsenic related genes could be found with different primer sets (Table 2). The presence of the different genes is indicated by shades of gray; absence by white.

Primers for the *arrA* gene retrieved amplicons from all samples (Fig. 5). In most cases, the three primer pairs tested produced amplicons. In the water samples, primers arrA1 and arrA2 always produced the same result (17 positives and two negatives). In the sediment samples, however, primer arrA1 was always positive while arrA2 had three negative results. Primer pair arrA3 was the one with the largest number of negatives (three water and four sediment samples) but in two water samples it was the only one with positive results. In summary, all samples were positive with at least one of the primer sets.

The results for the *arsC* gene were more complicated (Fig. 5). We tried three primer pairs from the literature and tested three new ones designed in the present study. Primer pair arsC-Grx-Sun targeted the genes of Enterobacteria, *Pseudomonas fluorescens* and *Acidiphilum multivorum* (Fig. 2), and it produced positive results only in the six water samples with the lowest As concentrations. Primer pair arsC-Grx-Saltikov (specific for *Shewanella*) did not produce clear results. Four samples showed a very faint band in the gels that was difficult to consider as positive. In one case, two bands appeared suggesting there was a non-specific amplification. Finally, there was one clear band in two more samples: the water sample with the lowest As concentration and the P4-04 sediment sample. In view of these ambiguous results, we cannot trust this primer pair. Primer pair arsC-Trx-Villegas was designed to target a group of Firmicutes genes and it was positive only in water sample P9. This sample produced amplicons with all the primer sets tested (Fig. 5).

The existence of *arsC* genes not targeted by the three primer sets described so far was a likely explanation for their failure to retrieve genes in Ascotán [19]. The phylogenetic tree built with the arsC sequences obtained from the completely sequenced genomes confirmed the occurrence of a third prokaryotic cluster of arsC sequences, in addition to the two prokaryotic clades proposed by Mukhopadhyay et al. (2002) [12]. The third cluster consisted mostly of Firmicutes, but was closer to the enterobacterial GRX than to the Firmicutes TRX cluster (Fig. 2).

The designed primer pairs arsC-Trx1a and arsC-Trx1b targeted essentially the same groups as arsC-Trx-Villegas (Fig. 2) which produced amplicons only in one sample (Fig. 5). However, we obtained positive amplification in most samples, except in two water samples (V-10_2006 and P6-05) and one sediment sample (P4-04), for the primers arsC-Trx1a. The primer set arsC-Trx1b was positive in five water and four sediment samples, one of each had not amplified with primer set arsC-Trx1a. The arsC-Trx1a and arsC-Trx1b sets designed to match with the first Firmicutes group were able to retrieve genes from most of the samples, including the ones with the higher As concentrations. However, when we used the primer set arsC-Trx2 designed to match the other Firmicutes group only nine water and eight sediment samples, spanning different arsenic concentrations, amplified. The samples with positive results using the primer set arsC-Trx2 also gave positive PCR with primers arsC-Trx1a and arsC-Trx1b. Overall, these results are consistent with Firmicutes being dominant in these samples (Figs. 3 and 4). Likewise, Firmicutes were the most common bacteria found in enrichment cultures from these samples [46]. In total, 86% of the samples showed positive PCR results with arr1 primers and arsC primers designed for the two Firmicutes clusters (Fig. 5).

In summary, the Enterobacteria related *arsC* genes were present in the water samples with the lowest As concentrations only, while the Firmicutes related genes could be found in samples spanning the whole range of arsenic concentrations evaluated in this study, although not in all the samples.

The fragments of arsC genes retrieved after sequencing of the PCR product of sample P9 using the primer pair arsC-Grx-Sun formed a clade within the Enterobacteria (Fig. 2). Meanwhile the sequences of the fragments obtained after the PCR of 10 samples −P405, P11, P604, P704, P806 P405, P604, QAB, P9 QJere− using the primer pair arsC-Trx1a and arsC-Trx1b were found within the Trx clade described before [12] (Fig. 2).

We tested two primer pairs targeting the *aioA1* and *aioA2* genes respectively. *aioA2* was amplified from most samples (fifteen water and seven sediments), while *aioA1* appeared in a subset of the samples positive for *aioA2*. Seven samples with very different As concentration did not produce any amplification. Finally, two primer sets were tested targeting the *acr3* As(III) transporter gene. acr3 2 showed positive results in seven water and five sediment samples, while acr3 1 only had one positive result.

## Discussion

### As(V) dissimilatory reduction

The capability to respire As(V) is widespread in different prokaryotic groups such as Proteobacteria, Firmicutes, Chrysiogenetes, Deinococcus-Thermus, Deferribacteres and Chrenarchaeota. Yet, all well studied As(V) respirers use homologs of the *arrAB* operon for this process. The *arrA* gene codes a respiratory arsenate reductase and *arrB* transfers electrons from the electron transport chain to the previous enzyme, thus obtaining energy from the electron transport. The ArrA proteins form a well-defined cluster within the family of DMSO reductases [5], [6]. Three different primer sets have been developed targeting the *arrA* gene. Malasarn et al. (2004) [6] developed the arrA1 set matching a diverse group of eight bacteria including Proteobacteria, Chrysiogenetes and Firmicutes (Table 2). With this primer set, these authors were able to retrieve *arrA* genes from Haiwee Reservoir [6] and Hollibaugh et al. (2006) [47] found the gene in Mono Lake. Lear et al. (2007) [24] used primer set arrA2 to detect the genes in mesocosms with sediments from contaminated aquifers. Finally, Kulp et al. (2006) [23] modified the arrA1 primers developing primer set arrA3 to include archaeal genes and retrieved sequences from Mono and Searles Lakes anoxic sediments. The latter primers produced longer amplicons and targeted a larger number of *arrA* genes than the arrA1 set (Table 2). We used the three primer sets trying to maximize the recovery of *arrA* genes. Surprisingly, primer set arrA3 had more negative results than arrA1 (Fig. S2), despite the fact that it was supposedly an improvement over the latter. These results could be understood if there are still many *arrA* genes in nature that have not yet been retrieved in sequence collections. Thus, the primers developed with just a few pure cultures are likely missing different *arrA* genes present in the environment.

At any rate, the interesting fact was that *arrA* genes were present in all samples (Fig. S2). A priori, we had expected this gene to be present at the higher end of the spectrum of As concentrations only, since dissimilatory reduction requires large concentrations of the electron acceptor [48]. Also, we were expecting it in the sediment samples where anaerobiosis is more likely. Yet, the gene was present throughout a range of six orders of magnitude of As concentrations and in all water samples with dissolved oxygen concentrations close to saturation. When most probable number estimates of arsenate reducing bacteria were carried out in a previous study, positive growth was found in all the sediment samples with high As concentrations (larger than 370 mg Kg^−1^), while all the water samples with As concentrations lower than 4 mg L^−1^ showed negative results [22]. At intermediate concentrations, some samples were positive and others negative. These results are consistent with As-respiring bacteria being present only at the highest As concentrations, particularly in the sediments. One possible explanation for the presence of this gene in aerobic environments is the recent discovery that the respiratory arsenate reductase may act in some cases as a bidirectional enzyme and the redox potential of the molybdenum center could play a role in determining the direction [26], [49], [50]. The expression of the related *arxA* gene (not targeted in this study) is only induced with arsenite and under anaerobic conditions [50]. Thus, the enzyme could be used in different ways in different environments. In addition, evidence has been reported of a wide distribution of anaerobic arsenite oxidation activity associated to *arx* genes (arx-based arsenotrophy) [26]. Further, the *arx* gene resembles the genes encoding the catalytic subunit of the ArrA [49] and ArxA has been established as a new clade of arsenite oxidases within the DMSO reductase family of molybdenum oxidoreductases [26]. However, the primers used to amplify *arr*A gene hit the *arx*A gene sequence of *Alkalilimnicola ehrlichii* MLHE-1 and only the forward primer used to amplify *arr*A gene partially hits the *arx*A gene sequence of *Ectothiorhodospira* sp. PHS-1.Therefore, a cross reaction is likely in view of the available arxA sequences in data bases.

### As(V) detoxifying reduction

The primers targeting the GRX class including enterobacterial gamma-Proteobacteria and *Shewanella* [19], [51] and the TRX class [20] (Table 2) retrieved few *arsC* genes from our samples (Fig. 5 and Fig. S2). arsC-Grx-Sun primers only found targets in the six water samples with the lowest As concentration and the arsC-Grx-Saltikov primers gave doubtful results. This is not surprising since the last pair was designed specifically for *Shewanella* and there is no reason why this bacterium should be present in Ascotán. Bacteria with arsC genes of the Grx-enterobacterial clade do not seem to tolerate As concentrations above 4 mg L^−1^. In agreement with this, Sun et al. (2004) [19] only found the *arsC* gene using general PCR after incubation, and they only detected it by real-time PCR in the natural samples from a contaminated mine soil. As concentrations in the studied sites were not reported, however, and comparison with ours is not possible. Likewise, the gene was not detected in a Cambodian contaminated sediment with 13.1 mg Kg^−1^ of arsenic [24]. These primers may not be the most efficient at retrieving arsC genes from different environments, likely because they target only a small group of bacteria. Obviously, the diversity of known *arsC* sequences restricts the detection of *arsC* genes when using probes designed from phylogenetically different organisms [19].

We were more intrigued by the results from the arsC-Trx-Villegas set. This primer set was designed targeting a group of *arsC* genes coding TRX-coupled enzymes [20], mostly from Firmicutes (Table 2). In the present study, only one sample was positive with this primer set (Fig. S2). This was unexpected because the DGGE and the clone library of the 16S rRNA gene from samples showed Firmicutes to be dominant members of the community (Figs. 3 and 4).

Jackson and Dugas (2003) [17] analyzed a larger data set of *arsC* sequences than that considered by Mulkophadhyay et al. (2002) [12] and concluded that the most likely explanation for the diversity of *arsC* genes was an early origin of the gene with diversification leading to the three main branches proposed by Mulkophadhyay et al. [12]. Several other branches with a small number of known sequences in the data bases are part of that diversity [12]. Their tree, in effect, showed several different clades in addition to the three proposed by Mulkophadhyay et al. (2002). Moreover, recently, a new family of *arsC* genes (the mycothiol (MSH)/mycoredoxin (Mrx)-dependent class) has been discovered in *Corynebacterium glutanicum* [14] and *Mycobacterium tuberculosis* [15].

Both the number and type (Trx or Grx) of *arsC* genes present in the genomes of prokaryotic organisms have been shown to impact their arsenic resistance level [16], [52]–[54]. The Trx reducing system has been reported to be the most efficient in arsenate decontamination [16]. In addition, many Trx-linked arsenate reductases have been found in low G + C Gram positive bacteria [7] and we have shown that these bacterial groups are predominant in arsenic impacted environments [46], [55]. Thus, the absence of Grx-associated *arsC* genes in the environments with intermediate and high concentrations of As found in the present study could be explained by the predicted lower efficiency of the reductases.

### As(III) oxidation

A large variety of genes involved in As(III) oxidation have been described [10], [56]. Lett et al. (2011) [25] attempted to unify the nomenclature, since many homologous genes had been named differently by different authors. We have followed their nomenclature here. The genes include two subunits of the arsenite oxidase enzyme (*aioA* and *aioB*), a sensor histidine kinase (*aioS*), a transcriptional regulator (*aioR*) and an oxyanion binding protein (*aioX*). Inskeep et al. (2007) [5] developed two sets of primers targeting the *aioA* gene (Table 2). They used seven phylogenetically distant bacteria for the design and tested the primers with a series of known As oxidizer pure cultures. These primer sets were then successfully used to retrieve *aioA* genes from different environments, including contaminated soils [57], geothermal springs [5] and a lake sediment. The As concentrations of these environments ranged between 0.01 and 1.74 µg L^−1^ As, at the lowermost end of the range of values considered in the present study. These primers were used to detect the presence of the gene in a number of pure cultures [57] finding the *aioA* gene only in five Proteobacteria out of 58 As-resistant bacteria tested. Campos et al. (2011) [58] also found this gene in bacteria isolated from Quebrada Camarones, a desert stream a few hundred kilometers from our studied sites. To our knowledge, these primers have not been tested in other natural environments.

Using these primers, we found 14 and 22 positive responses from primer sets aioA1 and aioA2, respectively (Fig. S2). When primer set aioA1 gave a positive response, the other set also gave a positive response. The positive amplifications were distributed throughout the range of As concentrations and both in water and sediment samples. This extends the range of concentrations where this gene has been found in environmental samples by four orders of magnitude [33]. Most of the samples that did not show the presence of this gene were from hot springs, both water and sediment samples, but we do not have enough data to test whether this is a robust characteristic or not.

### As(III) transport

There are two unrelated families of arsenite transporters in Bacteria. The *arsB* pump is associated with the *arsC* reductase. We decided not to look for the *arsB* transporter because it is most frequently associated with *arsC*, a gene we were already targeting it with six primer sets. We assumed a distribution similar to that of the *arsC* genes (see above). The last As related genes we tested were the As(III) transport genes *acr3*, of which two varieties have been identified. Achour et al. (2007) [29] designed two sets of primers acr3 1 and acr3 2 for those varieties and tested these primers plus a set of primers for the *arsB* gene with a series of As-resistant isolates from soil samples with different levels of arsenic contamination. They found that the *acr3* gene was more typical than *arsB* in those 41 strains. In addition, *arsB* was prevalent in Firmicutes and Gammaproteobacteria while *acr3-1* and *acr3-2* genes were more common in Actinobacteria and Alphaproteobacteria, respectively. Cai et al. (2009) [57] successfully used these primers to detect the genes in As-resistant bacteria isolated from contaminated soils. The *acr3-1* gene was present in 10 Gammaproteobacteria and two Actinobacteria, while the *acr3-2* gene was found in 21 isolates from Alpha-, Beta- and Gammaproteobacteria, seven in each. These primers with modifications have also been tested in samples taken from Rifle aquifer CO, USA, with As levels of 1.5 µM, and sequences from both subfamilies were recovered [59] With the acr3 1 primers we found amplification only in one sample from a hot spring at El Tatio. This result is consistent with the absence of Actinobacteria in water and sediment samples from Ascotán according to the 16S rRNA DGGE and gene library data (Figs. 3 and 4). With the acr3 2 set, however, we obtained amplification from seven water and five sediment samples (Fig. S2). Interestingly, the amplification results with the arsC-Trx2 and the acr3 2 sets of primers were coincident in more than 60% of the samples. This makes sense considering that the genomes of all the microorganisms used to design the arsC-Trx2 primers also contained the *acr3* gene, while this was not the case of the microorganisms used to design the arsC-Trx1a and arsC-Trx1b primer sets.

An increased number of positive amplification of As related genes was found in sediment samples compared to water samples. The analysis of the 16S rRNA genetic libraries from a sediment and a water sample (Fig. 4) indicated an S_Chao1_ index calculation of 590 for sediments and 42.5 for water samples (at 99% similarity). This higher diversity in sediment than in water samples has been found in similar environments [60]-[62]. Thus, the increased number of As processing genes in the sediments could be a consequence of the larger phylogenetic diversity of the sediment communities.

### Concluding remarks

Most As-related genes were widely distributed throughout the six orders of magnitude range of As concentrations obtained after analyzing very heterogeneous environments, such as shallow lagoons brines, sediments, hot springs, and salt deposits. This study is based on PCR and some concerns should be considered. Instead of using specific PCR primers with a given sequence, we tried to increase the primer coverage by using degenerate PCR primers, designed after an extensive GenBank search, to target the sequences present in such diverse environmental set. We were very careful to minimize the limitations to properly obtain specific PCR products. Priming conditions were optimized after testing the annealing temperatures coupled to amplicon cloning and sequencing. To what extent mismatches may have affected the efficiency of the PCR is however something unknown.The fact that some genes could not be amplified in some of the samples could be due to the environmental heterogeneity or to methodological limitations. The *arsC* gene, however, provided a rather clear pattern. The enterobacterial related variant of the gene was only present in the lower As concentrations, while the Firmicutes related gene variants were present in many different samples including those with the highest As concentrations. The *arsC* gene has experienced a large diversification, and apparently different bacterial lineages have inherited variants of the gene providing different degrees of protection against arsenic. The diversity of As processing genes found in the sediment samples was larger than in the water samples, in agreement with the larger phylogenetic bacterial diversity found in sediments than in water samples.

## Supporting Information

Figure S1 Primer targeting location on the arsenate cytoplasmic reductase genes (arsC) from Firmicutes phylum. A) Primer set arsC-Trx1a (black frames) and b (grey frames) and arsC sequences from *Bacillus thuringiensis, Bacillus cereus, Geobacillus kaustophilus, Bacillus clausii, Oceanobacillus iheyensis* and *Bacillus halodurans*; B) Primer set arsC-Trx2 (black frames) and arsC sequences from *Geobacillus thermoglucosidasius, Geobacillus* sp., *Alkaliphilus oremlandii* OhILAs and *Bacillus subtilis.*
(TIF)

Figure S2 Number of samples with PCR positive results for targeted genes using the previously described and newly designed sets of primers (Table 2).
(TIF)

Table S1 Blast output for sequenced DGGE bands from water an sediment samples in Salar de Ascotán, Tatio Geyser field and Salar de Atacama (Quebradas Aguas Blancas and Jere).
(PDF)

## References

[1] Smedley PL, Kinniburgh DG (2002) A review of the source, behaviour and distribution of arsenic in natural waters. Appl Geochem 17: 517–568.

[2] Ball P (2005) Arsenic-free water still a pipedream. Nature 436: 313. 10.1038/436313a 16034383

[3] Croal LR, Gralnick JA, Malasarn D, Newman DK (2004) The genetics of geochemistry. Annu Rev Genet 38: 175–202. 15568975 10.1146/annurev.genet.38.072902.091138

[4] Oremland RS, Stolz JF (2003) The ecology of arsenic. Science 300: 939–944. 12738852 10.1126/science.1081903

[5] Inskeep WP, Macur RE, Hamamura N, Warelow TP, Ward SA, et al (2007) Detection, diversity and expression of aerobic bacterial arsenite oxidase genes. Environ Microbiol 9: 934–943. 17359265 10.1111/j.1462-2920.2006.01215.x

[6] Malasarn D, Saltikov CW, Campbell KM, Santini JM, Hering JG, et al.. (2004) arrA Is a Reliable Marker for As(V) Respiration. Science 306: 455-. 10.1126/science.1102374 15486292

[7] Messens J, Silver S (2006) Arsenate reduction: thiol cascade chemistry with convergent evolution. J Mol Biol 362: 1–17. 16905151 10.1016/j.jmb.2006.07.002

[8] Marapakala K, Qin J, Rosen BP (2012) Identification of Catalytic Residues in the As(III) S-Adenosylmethionine Methyltransferase. Biochemistry-Us 51: 944–951. 10.1021/bi201500c PMC3431559 22257120

[9] Paez-Espino D, Tamames J, de Lorenzo V, Canovas D (2009) Microbial responses to environmental arsenic. Biometals 22: 117–130. 19130261 10.1007/s10534-008-9195-y

[10] Slyemi D, Bonnefoy V (2012) How prokaryotes deal with arsenic†. EMIR 4: 571–586. 10.1111/j.1758-2229.2011.00300.x 23760928

[11] Kleerebezem M, Boekhorst J, van Kranenburg R, Molenaar D, Kuipers OP, et al (2003) Complete genome sequence of Lactobacillus plantarum WCFS1. P Natl Acad Sci USA 100: 1990–1995. 10.1073/pnas.0337704100 PMC149946 12566566

[12] Mukhopadhyay R, Rosen BP, Phung LT, Silver S (2002) Microbial arsenic: from geocycles to genes and enzymes. FEMS Microbiol Rev 26: 311–325. 12165430 10.1111/j.1574-6976.2002.tb00617.x

[13] Zegers I, Martins JC, Willem R, Wyns L, Messens J (2001) Arsenate reductase from S. aureus plasmid pI258 is a phosphatase drafted for redox duty. Nat Struct Biol 8: 843–847. 11573087 10.1038/nsb1001-843

[14] Ordonez E, Van Belle K, Roos G, De Galan S, Letek M, et al (2009) Arsenate reductase, mycothiol, and mycoredoxin concert thiol/disulfide exchange. J Biol Chem 284: 15107–15116. 19286650 10.1074/jbc.M900877200 PMC2685692

[15] Van Laer K, Buts L, Foloppe N, Vertommen D, Van Belle K, et al.. (2012) Mycoredoxin-1 is one of the missing links in the oxidative stress defence mechanism of Mycobacteria. Mol Microbiol. 10.1111/mmi.12030 22970802

[16] Villadangos AF, Van Belle K, Wahni K, Tamu Dufe V, Freitas S, et al (2011) Corynebacterium glutamicum survives arsenic stress with arsenate reductases coupled to two distinct redox mechanisms. Mol Microbiol 82: 998–1014. 22032722 10.1111/j.1365-2958.2011.07882.x

[17] Jackson CR, Dugas SL (2003) Phylogenetic analysis of bacterial and archaeal arsC gene sequences suggests an ancient, common origin for arsenate reductase. BMC Evol Biol 3.. 10.1186/1471-2148-3-18 PMC183826 12877744

[18] Saltikov CW, Wildman RA, Newman DK (2005) Expression dynamics of arsenic respiration and detoxification in Shewanella sp strain ANA-3. J Bacteriol 187: 7390–7396. 16237022 10.1128/JB.187.21.7390-7396.2005 PMC1272973

[19] Sun YM, Polishchuk EA, Radoja U, Cullen WR (2004) Identification and quantification of arsC genes in environmental samples by using real-time PCR. J Microbiol Meth 58: 335–349. 10.1016/j.mimet.2004.04.015 15279938

[20] Villegas-Torres MF, Bedoya-Reina OC, Salazar C, Vives-Florez MJ, Dussan J (2011) Horizontal *arsC* gene transfer among microorganisms isolated from arsenic polluted soil. Int Biodeter Biodegr 65..

[21] Newman DK, Beveridge TJ, Morel FMM (1997) Precipitation of Arsenic trisulphide by *Desulfotomaculum aurpigmentum* . Appl Environ Microb 63: 2022–2028. 10.1128/aem.63.5.2022-2028.1997 PMC1389166 16535611

[22] Demergasso C, Chong G, Escudero L, Pueyo J, Pedrós-Alió C (2007) Microbial precipitation of arsenic sulfides in Andean salt flats. Geomicrobiol J 24: 111–123.

[23] Kulp TR, Hoeft SE, Miller LG, Saltikov C, Murphy JN, et al (2006) Dissimilatory arsenate and sulfate reduction in sediments of two hypersaline, arsenic-rich soda lakes: Mono and Searles Lakes, California. Appl Environ Microbiol 72: 6514–6526. 17021200 10.1128/AEM.01066-06 PMC1610296

[24] Lear G, Song B, Gault AG, Polya DA, Lloyd JR (2007) Molecular analysis of arsenate-reducing bacteria within Cambodian sediments following amendment with acetate. Appl Environ Microbiol 73: 1041–1048. 17114326 10.1128/AEM.01654-06 PMC1828664

[25] Lett MC, Muller D, Lievremont D, Silver S, Santini J (2012) Unified nomenclature for genes involved in prokaryotic aerobic arsenite oxidation. J Bacteriol 194: 207–208. 22056935 10.1128/JB.06391-11 PMC3256664

[26] Zargar K, Conrad A, Bernick DL, Lowe TM, Stolc V, et al (2012) ArxA, a new clade of arsenite oxidase within the DMSO reductase family of molybdenum oxidoreductases. Environ Microbiol 14: 1635–1645. 22404962 10.1111/j.1462-2920.2012.02722.x

[27] Wysocki R, Bobrowicz P, Ulaszewski S (1997) The Saccharomyces cerevisiae ACR3 gene encodes a putative membrane protein involved in arsenite transport. J Biol Chem 272: 30061–30066. 9374482 10.1074/jbc.272.48.30061

[28] Sato T, Kobayashi Y (1998) The ars operon in the skin element of Bacillus subtilis confers resistance to arsenate and arsenite. J Bacteriol 180: 1655–1661. 9537360 10.1128/jb.180.7.1655-1661.1998 PMC107075

[29] Achour AR, Bauda P, Billard P (2007) Diversity of arsenite transporter genes from arsenic-resistant soil bacteria. Diversity of arsenite transporter genes from arsenic-resistant soil bacteria 158: 128–137. 10.1016/j.resmic.2006.11.006 17258434

[30] Silver S, Phung LT (2005) Genes and enzymes involved in bacterial oxidation and reduction of inorganic arsenic. Appl Environ Microbiol 71: 599–608. 15691908 10.1128/AEM.71.2.599-608.2005 PMC546828

[31] Qin J, Rosen BP, Zhang Y, Wang G, Franke S, et al (2006) Arsenic detoxification and evolution of trimethylarsine gas by a microbial arsenite S-adenosylmethionine methyltransferase. Proc Natl Acad Sci USA 103: 2075–2080. 16452170 10.1073/pnas.0506836103 PMC1413689

[32] Stolz JF, Basu P, Santini JM, Oremland RS (2006) Arsenic and Selenium in Microbial Metabolism. Annu Rev Microbiol 60: 107–130. 16704340 10.1146/annurev.micro.60.080805.142053

[33] Quemeneur M, Cebron A, Billard P, Battaglia-Brunet F, Garrido F, et al (2010) Population structure and abundance of arsenite-oxidizing bacteria along an arsenic pollution gradient in waters of the upper isle River Basin, France. Appl Environ Microbiol 76: 4566–4570. 20453153 10.1128/AEM.03104-09 PMC2897427

[34] Handley KM, Héry M, Lloyd JR (2009) Redox cycling of arsenic by the hydrothermal marine bacterium Marinobacter santoriniensis. Environ Microbiol 11: 1601–1611. 19226300 10.1111/j.1462-2920.2009.01890.x

[35] Smith AH, Arroyo AP, Mazumder DN, Kosnett MJ, Hernandez AL, et al (2000) Arsenic-induced skin lesions among Atacameno people in Northern Chile despite good nutrition and centuries of exposure. Environ Health Perspect 108: 617–620. 10903614 10.1289/ehp.00108617 PMC1638201

[36] Song B, Chyun E, Jaffe PR, Ward BB (2009) Molecular methods to detect and monitor dissimilatory arsenate-respiring bacteria (DARB) in sediments. FEMS Microbiol Ecol 68: 108–117. 19291024 10.1111/j.1574-6941.2009.00657.x

[37] Hamamura N, Macur RE, Korf S, Ackerman G, Taylor WP, et al (2009) Linking microbial oxidation of arsenic with detection and phylogenetic analysis of arsenite oxidase genes in diverse geothermal environments. Environ Microbiol 11: 421–431. 19196273 10.1111/j.1462-2920.2008.01781.x

[38] Rhine ED, Ni Chadhain SM, Zylstra GJ, Young LY (2007) The arsenite oxidase genes (aroAB) in novel chemoautotrophic arsenite oxidizers. Biochem Bioph Res CO 354: 662–667. 10.1016/j.bbrc.2007.01.004 17257587

[39] Macur RE, Jackson CR, Botero LM, McDermott TR, Inskeep WP (2004) Bacterial populations associated with the oxidation and reduction of arsenic in an unsaturated soil. Environ Sci Technol 38: 104–111. 14740724 10.1021/es034455a

[40] Butcher BG, Deane SM, Rawlings DE (2000) The chromosomal arsenic resistance genes of Thiobacillus ferrooxidans have an unusual arrangement and confer increased arsenic and antimony resistance to Escherichia coli. Appl Environ Microbiol 66: 1826–1833. 10788346 10.1128/aem.66.5.1826-1833.2000 PMC101419

[41] Mardones-Pérez L (1997) Flux et évolution des solutions salines dans les systemes hydrologiques des salars dAscotán et dAtacama. [Theses de Doctorat en Sciences de la Terre]. Paris: Université Paris-Sud/Orsay. 203 p.

[42] Risacher F, Alonso H, Salazar C (1999) Geoquímica de aguas en cuencas cerradas: I, II y III Regiones–Chile. Technical Report S.I.T. No. 51. Santiago, Chile.: Convenio de Cooperación DGA, UCN, IRD.

[43] Demergasso C, Escudero L, Casamayor EO, Chong G, Balague V, et al (2008) Novelty and spatio-temporal heterogeneity in the bacterial diversity of hypersaline Lake Tebenquiche (Salar de Atacama). Extremophiles 12: 491–504. 18347752 10.1007/s00792-008-0153-y

[44] Jeanmougin F, Thompson JD, Gouy M, Higgins DG, Gibson TJ (1998) Multiple sequence alignment with Clustal X. . Trends Biochem Sci 23: 403–405. 9810230 10.1016/s0968-0004(98)01285-7

[45] Altschul SF, Madden TL, Schaffer AA, Zhang JH, Zhang Z, et al (1997) Gapped BLAST and PSI-BLAST: a new generation of protein database search programs. Nucleic Acids Res 25: 3389–3402. 9254694 10.1093/nar/25.17.3389 PMC146917

[46] Lara J, González LE, Ferrero M, Díaz GC, Pedrós-Alió C, et al (2012) Enrichment of arsenic transforming and resistant heterotrophic bacteria from sediments of two salt lakes in Northern Chile. Enrichment of arsenic transforming and resistant heterotrophic bacteria from sediments of two salt lakes in Northern Chile 16: 523–538. 10.1007/s00792-012-0452-1 22555750

[47] Hollibaugh JT, Budinoff C, Hollibaugh RA, Ransom B, Bano N (2006) Sulfide oxidation coupled to arsenate reduction by a diverse microbial community in a Soda Lake. Appl Environ Microb 72: 2043–2049. 10.1128/AEM.72.3.2043-2049.2006 PMC1393214 16517653

[48] Newman DK, Kennedy EK, Coates JD, Ahmann D, Ellis DJ, et al (1997) Dissimilatory arsenate and sulfate reduction in *Desulfotomaculum auripigmentum* sp. nov. Arch Microbiol 168: 380–388. 9325426 10.1007/s002030050512

[49] Richey C, Chovanec P, Hoeft SE, Oremland RS, Basu P, et al (2009) Respiratory arsenate reductase as a bidirectional enzyme. Biochem Biophys Res Commun 382: 298–302. 19285953 10.1016/j.bbrc.2009.03.045

[50] Zargar K, Hoeft S, Oremland R, Saltikov CW (2010) Identification of a Novel Arsenite Oxidase Gene, arxA, in the Haloalkaliphilic, Arsenite-Oxidizing Bacterium Alkalilimnicola ehrlichii Strain MLHE-1. J Bacteriol 192: 3755–3762. 20453090 10.1128/JB.00244-10 PMC2897359

[51] Saltikov CW, Cifuentes A, Venkateswaran K, Newman DK (2003) The ars detoxification system is advantageous but not required for As(V) respiration by the genetically tractable Shewanella species strain ANA-3. Appl Environ Microb 69: 2800–2809. 10.1128/AEM.69.5.2800-2809.2003 PMC154534 12732551

[52] Li X, Krumholz LR (2007) Regulation of arsenate resistance in Desulfovibrio desulfuricans G20 by an arsRBCC operon and an arsC gene. J Bacteriol 189: 3705–3711. 17337573 10.1128/JB.01913-06 PMC1913334

[53] Achour-Rokbani A, Cordi A, Poupin P, Bauda P, Billard P (2010) Characterization of the ars gene cluster from extremely arsenic-resistant Microbacterium sp. strain A33. Appl Environ Microbiol 76: 948–955. 19966021 10.1128/AEM.01738-09 PMC2813031

[54] Cuebas M, Villafane A, McBride M, Yee N, Bini E (2011) Arsenate reduction and expression of multiple chromosomal ars operons in Geobacillus kaustophilus A1. Microbiology+ 157: 2004–2011. 21511766 10.1099/mic.0.048678-0

[55] Escudero LV (2009) Diversity of arsenic genes involved in arsenic cycle in culture and environmental sample [PhD Thesis]. Barcelona, España: Universidad Autónoma de Barcelona.

[56] Silver S, Phung LT (2005) Novel expansion of living chemistry or just a serious mistake? FEMS Microbiol Lett 315: 79–80. 10.1111/j.1574-6968.2010.02202.x 21232070

[57] Cai L, Liu G, Rensing C, Wang G (2009) Genes involved in arsenic transformation and resistance associated with different levels of arsenic-contaminated soils. BMC Microbiol 9. 10.1186/1471-2180-9-4 PMC2631446 19128515

[58] Campos VL, Leon C, Mondaca MA, Yanez J, Zaror C (2011) Arsenic Mobilization by Epilithic Bacterial Communities Associated with Volcanic Rocks from Camarones River, Atacama Desert, Northern Chile. Arch Environ Con Tox 61: 185–192. 10.1007/s00244-010-9601-7 20859623

[59] Giloteaux L, Holmes DE, Williams KH, Wrighton KC, Wilkins MJ, et al (2013) Characterization and transcription of arsenic respiration and resistance genes during in situ uranium bioremediation. ISME J 7: 370–383. 23038171 10.1038/ismej.2012.109 PMC3554400

[60] Dorador C (2007) Microbial communities in high altitude altiplanic wetlands in northern Chile: phylogeny, diversity and function. [PhD]. Kiel: Christian-Albrechts-Universität. 170 p.

[61] Dorador C, Meneses D, Urtuvia V, Demergasso C, Vila I, et al (2009) Diversity of Bacteroidetes in high-altitude saline evaporitic basins in northern Chile. J Geophys Res-Biogeo 114: G00D05.

[62] Jiang H, Dong H, Zhang G, Yu B, Chapman L, et al (2006) Microbial diversity in water and sediment of Lake Chaka, an athalassohaline lake in northwestern China. Appl Environ Microbiol 72: 3832–3845. 16751487 10.1128/AEM.02869-05 PMC1489620

