# Supplementary figures and images for: Distribution of Microbial Arsenic Reduction, Oxidation and Extrusion Genes along a Wide Range of Environmental Arsenic Concentrations
Source: PLoS One. 2013 Oct 31;8(10):e78890. doi: 10.1371/journal.pone.0078890 (PMC3815024; doi:10.1371/journal.pone.0078890)

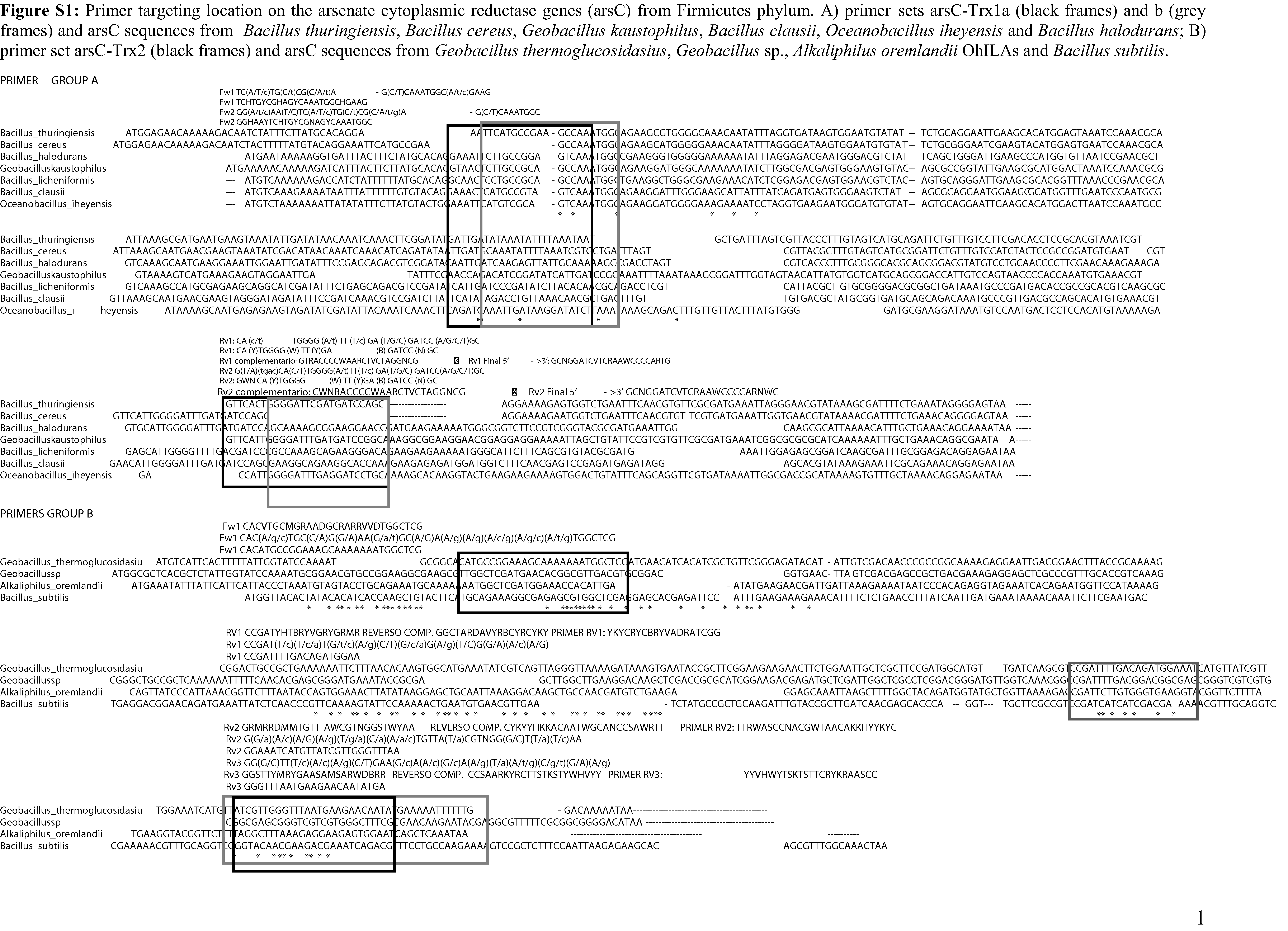

Supplement: Figure S1 — Primer targeting location on the arsenate cytoplasmic reductase genes (arsC) from Firmicutes phylum. A) Primer set arsC-Trx1a (black frames) and b (grey frames) and arsC sequences from Bacillus thuringiensis, Bacillus cereus, Geobacillus kaustophilus, Bacillus clausii, Oceanobacillus iheyensis and Bacillus halodurans; B) Primer set arsC-Trx2 (black frames) and arsC sequences from Geobacillus thermoglucosidasius, Geobacillus sp., Alkaliphilus oremlandii OhILAs and Bacillus subtilis. (TIF) [file pone.0078890.s001.tif]
