# Supplementary material for: Distribution of Microbial Arsenic Reduction, Oxidation and Extrusion Genes along a Wide Range of Environmental Arsenic Concentrations
Source: PLoS One. 2013 Oct 31;8(10):e78890. doi: 10.1371/journal.pone.0078890 (PMC3815024; doi:10.1371/journal.pone.0078890)
Supplement: Table S1 — Blast output for sequenced DGGE bands from water an sediment samples in Salar de Ascotán, Tatio Geyser field and Salar de Atacama (Quebradas Aguas Blancas and Jere). (PDF) [file pone.0078890.s003.pdf]

Table S1: Blastoupt for sequenced DGGE bands from water an sediment samples in Salar de Ascotan, Tatio Gyser field and Salar de Atacama (Quebradas Aguas Blancas and Jere).

Sample	*Lenght*	*Accession Number*	*Phylogenetic group*	*Closest relative*	*Accession Number*	*% Similarity*
**2004**						
P1-04B1	430	-	*Cyanobacteria*	Uncultured cyanobacterium clone SIMO-2257	AY711623	97
P6-04B2	461	AB844273	*Algas*	Uncultured phototrophic eukaryote clone PM2-29	EF215817	96
P1-04B3	539	AB844274	*Betaproteobacteria*	*Polynucleobacter sp.* SUWAF016	AB447552	98
P6-04B4	387	AB844275	*Bacteroidetes*	*Psychroflexus sp.* YIM C238	AF513434	89
P1-04B5	539	AB844276	*Betaproteobacteria*	*Polynucleobacter sp.* SUWAF016	AB447552	99
P1-04B6	483	AB844277	*Betaproteobacteria*	Uncultured beta proteobacterium clone PRD18D11	AY948031	98
P1-04B7	538	AB844278	*Betaproteobacteria*	*Polynucleobacter sp.* SUWAF016	AB447552	99
P1-04B8	492	AB844279	*Alphaproteobacteria*	Uncultured Rhodobacter sp. clone KS-13	EU809813	96
P7-04B13	509	AB844280	*Alphaproteobacteria*	Uncultured Rhodobacteraceae bacterium,isolate EG7	AM691097	89
P7-04B14	536	AB844281	*Firmicutes*	Uncultured firmicute isolate ikaite un-c22	AJ431344	87
P2-04B15	552	AB844282	*Bacteroidetes*	Flavobacteriaceae bacterium CNU041	EF217417	95
P2-04B16	543	AB844283	*Bacteroidetes*	Uncultured bacterium clone Hot Creek 2	AY168735	89
P1-04B21	533	AB844284	*Alphaproteobacteria*	*Loktanella sp. MOLA 317*	AM945550	87
P2-04B22	524	AB844285	*Alphaproteobacteria*	Uncultured alpha proteobacterium clone JL-WNPG-T46	FJ203409	85
P6-04B24	476	-	*Alphaproteobacteria*	Uncultured alpha proteobacterium clone T66ANG3	AJ633984	88
P7-04B26	365	AB844286	*Firmicutes*	Uncultured Natronoanaerobium, clone AS-P4-Sed-42	AJ940551	94
P2-04B28	404	AB844287	*Alphaproteobacteria*	*Loktanella vestfoldensis strain LMG22003*	DQ915611	92
**2005**						
P6-05B1	539	AB844288	*Gammaproteobacteria*	*Halothiobacillus* sp. NP36	EU196319	96
P6-05B2	540	AB844289	*Betaproteobacteria*	*Thiobacillus sp.* EBD bloom	DQ218323	91
P6-05B3	540	AB844290	*Gammaproteobacteria*	*Halothiobacillus* sp. NP36	EU196319	92
P4-05B4	484	-	*Gammaproteobacteria*	Uncultured gamma proteobacterium clone WN-FSB-209	DQ432134	77
P3-05B5	511	AB844291	*Deltaproteobacteria*	Uncultured Desulfotignum sp. clone A15-30-22	AJ966329	85
P4-05B6	548	AB844292	*Firmicutes*	*Halanaerobium* sp. AN-BI5B	AM157647	91
P4-05B10	517	AB844293	*Firmicutes*	Uncultured firmicute isolate ikaite un-c22	AJ431344	96
P4-05B11	543	AB844294	*Deltaproteobacteria*	*Desulfohalobium utahense* strain EtOH3	DQ067421	94
P4-05B20	538	AB844295	*Gammaproteobacteria*	*Halothiobacillus* sp. NP36	EU196319	95
P3-05B21	454	AB844296	*Cyanobacteria*	*Leptolyngbya* sp. 0BB19S12	AJ639895	74
**2006**						
P9-B-1	419	AB844318	*Firmicutes*	*Fusibacter* sp. enrichment culture clone 22-7A 16S	EU517558	97
P9-B-2	410	AB844319	*Firmicutes*	*Fusibacter* sp. enrichment culture clone 22-7A 16S	EU517558	75
P9-B-3	427	AB844320	*Firmicutes*	*Fusibacter* sp. enrichment culture clone 22-7A 16S	EU517558	96
**2007**						
SL-3-B1	525	AB844297	*Dienococus-thermus*	*Thermu* s W28 A.1 DNA fragment	L10068	96
SL-3-B2	524	AB844298	*Dienococus-thermus*	Uncultured Thermus sp. clone bacteriap1	AF402971	95
SL-3-B3	524	AB844299	*Dienococus-thermus*	*Thermus* W28 A.1 DNA fragment	L10068	96
SL-3-B4	530	AB844300	*Dienococus-thermus*	*Thermus* W28 A.1 DNA fragment	L10068	98
SL-1-B5	510	AB844301	*chloroplast*	*Galdieria maxima strain* IPPAS P507	AY391361	82
SL-1-B6	538	AB844302	*Betaproteobacteria*	*Thiomonas* intermedia	AY455809	93
SL-3-B7	543	AB844303	*Firmicutes*	Uncultured Sulfobacillus sp. clone K6-C156	EF612382	92
P11-B9	520	AB844304	*Alphaproteobacteria*	*Ruegeria sp* . 3X/A02/236	AY576770	80
V4-B10	428	AB844305	*Alphaproteobacteria*	Uncultured bacterium clone PB_B7	EF429656	79
P10-B12	533	AB844306	*Bacteroidetes*	*Flavobacterium bacterium 4*	FJ152552	96
P11-B18	543	AB844307	*Firmicutes*	*Anoxybacillus flavithermus* clone LK4	AJ810551	93
P11-B19	508	AB844308	*Alphaproteobacteria*	*Brevundimonas* sp. WPCB153	EU880921	90
V4-B22	428	AB844309	*Alphaproteobacteria*	Uncultured bacterium clone MQ_B15	EF429637	78
P11-B23	516	AB844310	*Alphaproteobacteria*	*Loktanella rosea* isolate IMCC1504	EU687492	94
V10-B24	553	AB844311	*Gammaproteobacteria*	Uncultured gamma proteobacterium clone: AN009	AJ313020	89
V4-B25	423	AB844312	*Alphaproteobacteria*	Uncultured bacterium clone LB_B4	EF429642	81
Q.Blanca-B-A	541	AB844313	*Gammaproteobacteria*	Uncultured gamma proteobacterium	AM116729	94
Q.Blanca-B-B	514	AB844314	*Alphaproteobacteria*	Unidentified alpha proteobacterium	AB002654	93
Q.Blanca-B-C	514	AB844315	*Alphaproteobacteria*	*Ruegeria* sp. 3X/A02/236 16S	AY576770	92
Q.Jere-B-D	462	AB844316	*Alphaproteobacteria*	Unidentified alpha proteobacterium	AB002654	93
Q.Jere-B-E	538	AB844317	*Beta proteobacterium*	*Beta proteobacterium Wuba139*	AF336363	87
